# High Response CO Sensor Based on a Polyaniline/SnO_2_ Nanocomposite

**DOI:** 10.3390/polym11010184

**Published:** 2019-01-21

**Authors:** Kai-Syuan Jian, Chi-Jung Chang, Jerry J. Wu, Yu-Cheng Chang, Chien-Yie Tsay, Jing-Heng Chen, Tzyy-Leng Horng, Gang-Juan Lee, Lakshmanan Karuppasamy, Sambandam Anandan, Chin-Yi Chen

**Affiliations:** 1 Department of Materials Science and Engineering, Feng Chia University, Taichung 407, Taiwan; seed111713@gmail.com (K.-S.J.); yuchchang@fcu.edu.tw (Y.-C.C.); cytsay@fcu.edu.tw (C.-Y.T.); 2 Department of Chemical Engineering, Feng Chia University, Taichung 407, Taiwan; changcj@fcu.edu.tw; 3 Department of Environmental Engineering and Science, Feng Chia University, Taichung 407, Taiwan; jjwu@fcu.edu.tw (J.J.W.); leeganjiuan@gmail.com (G.-J.L.); lksamylaksh@gmail.com (L.K.); 4 Department of Photonics, Feng Chia University, Taichung 407, Taiwan; jhchen@fcu.edu.tw; 5 Department of Applied Mathematics, Feng Chia University, Taichung 407, Taiwan; tlhorng@math.fcu.edu.tw; 6 Nanomaterials and Solar Energy Conversion Laboratory, Department of Chemistry, National Institute of Technology, Trichy 620015, India; sanand99@yahoo.com

**Keywords:** polyaniline, tin oxide, CO gas sensor, sol-gel, low operating temperature

## Abstract

A polyaniline (PANI)/tin oxide (SnO_2_) composite for a CO sensor was fabricated using a composite film composed of SnO_2_ nanoparticles and PANI deposition in the present study. Tin oxide nanoparticles were synthesized by the sol-gel method. The SnO_2_ nanoparticles provided a high surface area to significantly enhance the response to the change in CO concentration at low operating temperature (<75 °C). The excellent sensor response was mainly attributed to the relatively good properties of PANI in the redox reaction during sensing, which produced a great resistance difference between the air and CO gas at low operating temperature. Therefore, the combination of *n*-type SnO_2_ nanoparticles with a high surface area and a thick film of conductive PANI is an effective strategy to design a high-performance CO gas sensor.

## 1. Introduction

Carbon monoxide (CO) is a colorless, odorless, tasteless, but flammable and poisonous gas that is a product of internal combustion engines and the incomplete combustion of some types of organic matter. This toxic and hazardous gas can cause death at very low concentrations and serious long-term health problems or severe explosions at high concentrations in the atmosphere [1]. Therefore, the detection and monitoring of CO concentrations are crucial for poisoning prevention and environmental safety.

A semiconductor gas sensor, composed of a nanostructured metal oxide with an extremely high specific surface area, can provide a high sensing reaction involving either oxidation or reduction between the target gas and charged oxygen adsorbed on the surface. The adsorption or desorption of the target gas on the surface of the metal oxide changes either the conductivity or resistivity, which can be measured with electronic circuitry according to a known baseline value. Semiconductor gas sensors can achieve high sensitivity and rapid response [2] and have a relatively simple structure, so they are relatively inexpensive to manufacture due to their simplicity and scalability. 

Among the nanostructured metal oxides, zinc oxide (ZnO) [3], titanium oxide (TiO_2_) [4], gallium oxide (Ga_2_O_3_) [5], cerium oxide (CeO_2_) [6], and tin oxide (SnO_2_) [7,8,9] have been widely applied in CO gas sensors [1]. In recent years, tetragonal SnO_2_, an *n*-type semiconducting oxide, has been intensively investigated as a promising material due to its high sensitivity and selectivity to a wide range of CO gas concentrations at moderate operating temperature [10]. Numerous studies have demonstrated that SnO_2_ can be synthesized with nanostructures such as nanoparticles [11,12], nanotubes [13], nanorods [14], nanowires [15], and nanosheets [2] by various processes. However, the operating temperature of the SnO_2_ gas sensor is rather high (300 to 500 °C [16]). The operating temperature of the gas sensor tends to decrease when the dimensions of SnO_2_ are reduced to the nanoscale, which prevents the reduction of both the surface area and the catalytic properties of the material at high operating temperature [17]. 

Polyaniline (PANI) is an intrinsically conducting polymer (ICP) possessing the advantages of modifiable electrical conductivity, good environmental stability, inexpensive monomers, and ease of synthesis. Due to its good sensitivity towards CO gas at room temperature, it has been investigated for combination with metal oxide to form nanocomposites [18]. Although several studies have demonstrated that the sensing temperature of SnO_2_ powder coatings can be lowered by many means such as doping with noble metals [19,20] or PANI [21,22,23], and deposition onto various nanostructured templates [24,25], for application in gas sensors, to the best of our knowledge, the sol-gel SnO_2_ particle coating has not yet been drop-coated directly with various amounts of PANI for the optimization of low-temperature CO sensing performance. 

In the present study, undoped SnO_2_ nano-powder was synthesized by the sol-gel process. Subsequently, the powder was screen-printed onto a Pt-electrode coated Al_2_O_3_ substrate. After heat treatment, different amounts of PANI solution were deposited onto the surface of the SnO_2_ particles to fabricate a CO sensor. The structural properties of the sol-gel derived SnO_2_ powder and the screen-printed SnO_2_ coating with various amounts of PANI deposition were characterized by electron microscopy and x-ray diffractometry. The electrical resistivities of the SnO_2_ powder coatings with and without PANI deposition were measured under various CO concentrations as a function of operating temperature. The sensing behavior including the sensor response of the powder coatings to changes in CO concentration was investigated as a function of the PANI coating.

## 2. Experimental Procedures

The CO sensing properties of an undoped SnO_2_ coating with and without PANI deposition were investigated in the present study. The SnO_2_ powder was synthesized from tin (II) chloride dihydrate (TCD) and ethanol by the sol-gel process. The chemical formula of TCD is SnCl_2_·2H_2_O (98% purity, Macron Fine Chemicals, Radnor, PA, USA), and the molecular weight of reagent-grade TCD is 225.65. The TCD was dissolved in ethanol to form a precursor solution at room temperature. After stirring, aqueous ammonia was slowly added into the solution with continuous stirring until the solution precipitated to form a gel. The colloidal solution (gel) was centrifuged and rinsed with deionized water and ethanol several times. The nanosized SnO_2_ powder was then obtained by oven drying and heat treatment in a tubular furnace at 80 °C for 24 h and 400 °C in air for 2 h. Subsequently, the CO sensor was prepared by screen printing the sol-gel SnO_2_ powder onto a Pt-electrode-printed alumina substrate. Single interdigital structures of the Pt electrode were designed for the sensing measurements. The SnO_2_ powder paste was mixed with organics to create a paste. The details of the interdigital electrode configuration and paste composition can be found elsewhere [26,27]. Then, the screen-printed SnO_2_ film was heat-treated at 500 °C for 2 h in air to remove the residual organics and confirm the formation of the oxide coating. 

Nano-scaled PANI was synthesized from an aqueous solution of aniline (ANI) by the template-free method with ammonium peroxydisulfate (APS) as an oxidant [28]. The chemical formulas of ANI and APS are C_6_H_7_N (99.0% purity, Sigma Chemical Co., St. Louis, MO, USA) and (NH_4_)_2_S_2_O_8_, (98% purity, Alfa Aesar, Heysham, Lancashire, UK), respectively. Both were reagent grade. The molecular weights of ANI and APS are 93.13 and 228.19, respectively. First, 1.0 mL ANI was dissolved in 50 mL deionized water with stirring using 1.0 mL Triton-X as a dispersant at room temperature. Before polymerization, 1 M hydrochloride acid (HCl) was added dropwise into aniline monomer solutions at a pH value of <2. The precooled 50 mL of aqueous APS (0.25 mol/L) was added to the above solution and reacted in an ice bath below 15 °C for 4 hours. The resulting PANI precipitate was isolated by centrifugation and then washed with deionized water and ethanol several times until the suspension was completely colorless. Finally, the product was dried in a vacuum oven at 80 °C for 36 h. 

The SnO_2_/PANI sensor was prepared by drop-coating the suspension of PANI onto the surface of the heat-treated SnO_2_ coating. PANI was first dispersed in tetrahydrofuran (THF) to create a coating suspension. Then, the resulting solution was drop-coated onto the surface of the SnO_2_ coating using a micropipette (PR-1000, Rainin Instrument, LLC., Oakland, CA, USA) with weight ratios of PANI/SnO_2_ of 45/55 and 55/45 (45 and 55 wt % PANI-deposited SnO_2_). After the resulting composite sensor was dried at room temperature for 1 h, the operating temperature of the composite sensor was controlled by a Pt-electrode (printed on the back side) connected to a temperature/voltage controller. The electrical resistances of the SnO_2_ coating with and without PANI deposition were recorded in a homemade chamber as a function of temperature upon exposure to various concentrations of CO gas by a data acquisition apparatus (Agilent 34970A, Santa Clara, CA, USA). The sensor response of CO was defined as the ratio of *R*_air_/*R*_gas_, where *R*_air_ is the average sensor resistance in clean air and *R*_gas_ is the average sensor resistance during CO exposure upon stabilization of the response. 

The microstructures of the as-prepared powders and heat-treated thick films were observed using high resolution transmission electron microscopy (TEM, JEM-2010, JEOL, Tokyo, Japan) and field emission scanning electronic microscopy (FE-SEM, JSM-7800F, JEOL, Tokyo, Japan). The materials were identified by x-ray diffractometry using Cu*Kα* radiation (XRD, D8 Discover, Bruker Karlsruhe, Germany) and Fourier-transform infrared spectroscopy (FTIR, FT-IR Spectrometer Frontier, Perkin Elmer, Waltham, MA, USA). The specific surface areas of the powders were estimated by the BET (Brunauer–Emmer–Teller) method from nitrogen adsorption/desorption isotherm data on a constant-volume adsorption apparatus (SA-9600 Series, HORIBA, Kyoto, Japan). 

## 3. Results and Discussion

**Figure 1:**
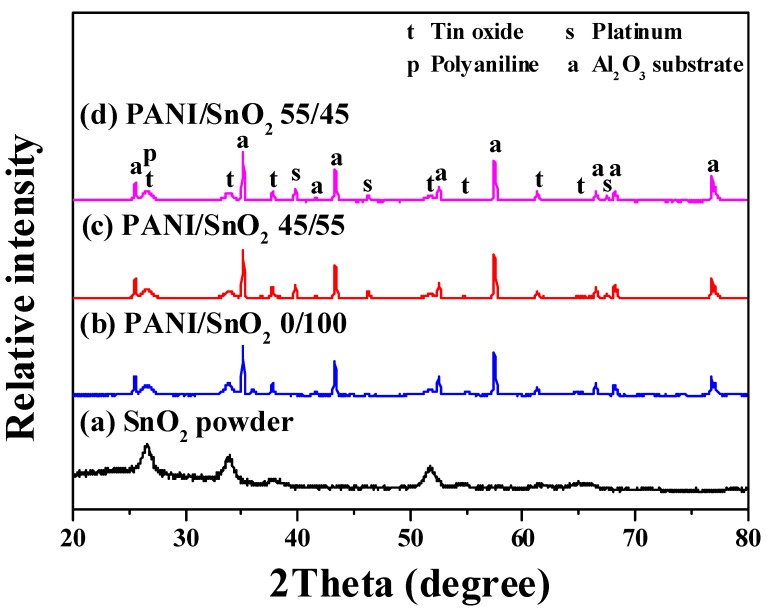
XRD patterns of the (**a**) as-synthesized SnO_2_ powder and screen-printed SnO_2_ powder coatings (**b**) without and with (**c**) 45 and (**d**) 55 wt % PANI depositions.

In the present study, the sol-gel synthesized SnO_2_ powder was screen-printed onto the substrate, after which different amounts of PANI were deposited for use in the CO sensor. Figure 1 shows the XRD patterns of the as-synthesized SnO_2_ powder and screen-printed SnO_2_ powder coatings without and with PANI deposition. The powder without PANI deposition, curve (a), was identified from the diffraction peaks as a tetragonal SnO_2_ phase, in accordance with JCPDS file No. 77-0448. The patterns of SnO_2_ powders with PANI deposition, curves (b–d), were identified as a mixture of SnO_2_ and PANI phases, revealing no peaks but those of the SnO_2_ and Al_2_O_3_ substrate. This implied that a sol-gel powder of high purity was obtained and confirmed that the PANI had been well-deposited onto the surface of the SnO_2_ powder by the drop coating method. Furthermore, the broad peaks of SnO_2_ in all of the patterns indicated that the powder and powder coatings were nanocrystalline. The crystallite sizes of the SnO_2_ powder (curve (a)) and coatings with 0, 45, and 55 wt % PANI depositions (curves (b–d)) were calculated to be ca. 6, 7, 7, and 7 nm by Scherrer’s formula, respectively

(1) $$d=\frac{0.9\lambda }{B\cos\theta }$$

where *d*, *λ*, *B*, and *θ* are the mean crystallite size, x-ray wavelength, full width (in radian) at half the maximum intensity of the diffraction peak, and peak diffraction angle, respectively. In this study, each crystallite size was calculated from three diffraction peaks at ca. 26.5°, 34°, and 52° in each pattern. No obvious changes in the crystallite size of the SnO_2_ coatings were found when different amounts of PANI were deposited. Such relatively small crystallite sizes of the sol-gel SnO_2_ were thus expected to provide a high sensing performance of the CO gas sensor. 

**Figure 2:**
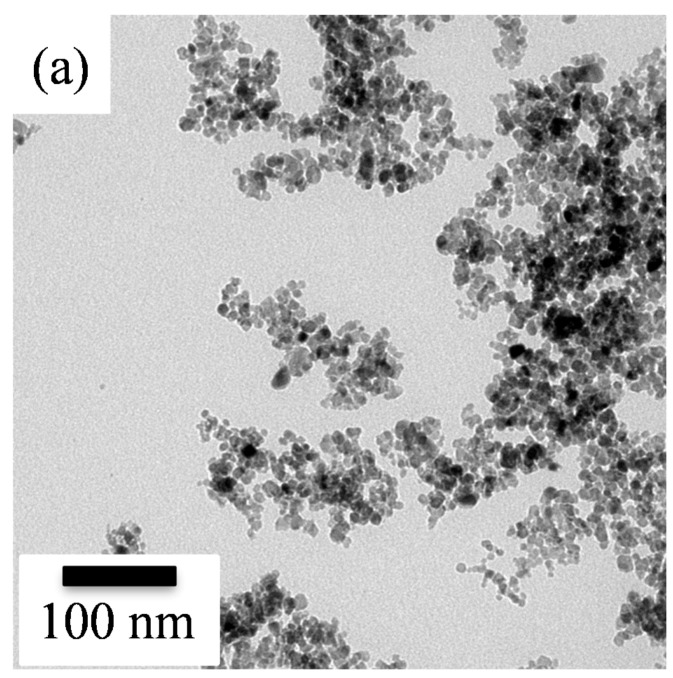

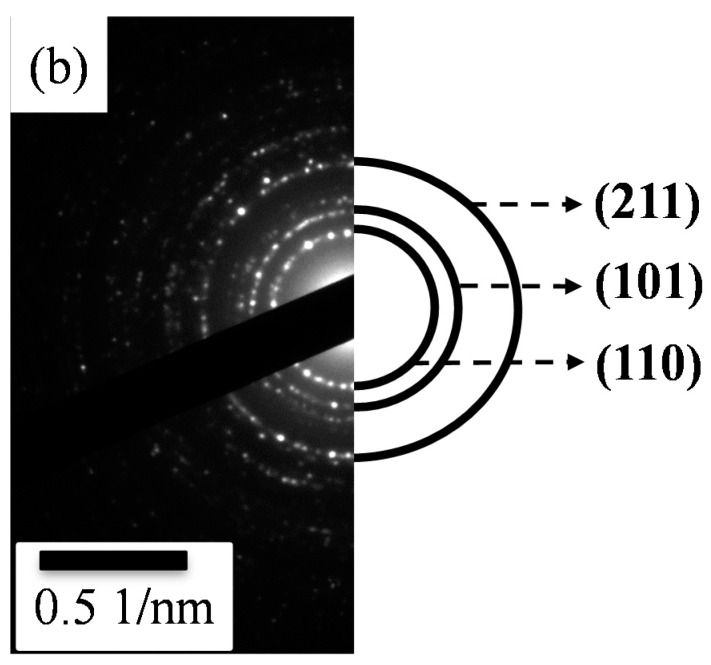
(**a**) TEM image and (**b**) its corresponding SAED patterns of sol-gel SnO_2_ nanoparticles.

The detailed structural characteristics of the resulting SnO_2_ powder were further investigated using transmission electron microscopy. Figure 2 shows the TEM images and their corresponding selected area electron diffraction (SAED) patterns of the sol-gel SnO_2_ nanoparticles without and with PANI deposition. The bright field image (Figure 2a) showed that the undeposited SnO_2_ powder was composed of clustered nanoparticles, revealing a rounded shape with an extremely small mean diameter of about 7 ± 2 nm. Meanwhile, according to the SAED pattern of the undeposited SnO_2_ shown in Figure 2b, a set of dashed circular rings indicated a nanocrystalline structure. The dashed circular rings obtained were respectively due to the diffraction of electrons from indexed (110), (101), and (211) planes of tetragonal SnO_2_. Furthermore, the surface area of the sol-gel SnO_2_ powders was ca. 45 m^2^/g. The nanocrystalline SnO_2_ powder was thus thought to provide a larger number of available active sites for sensing target gases, which in turn would provide better sensing performance. 

**Figure 3:**
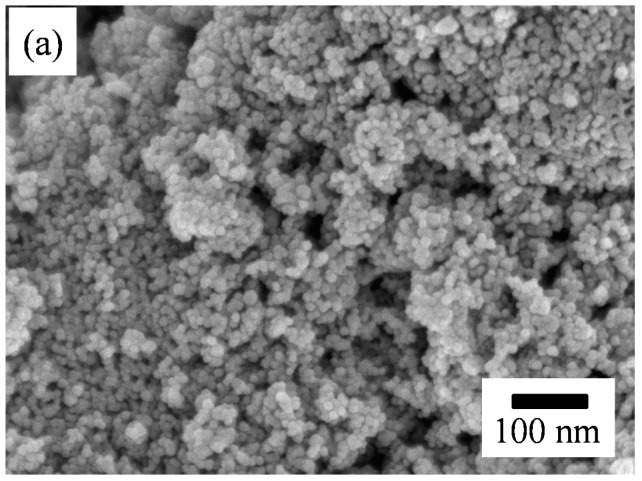

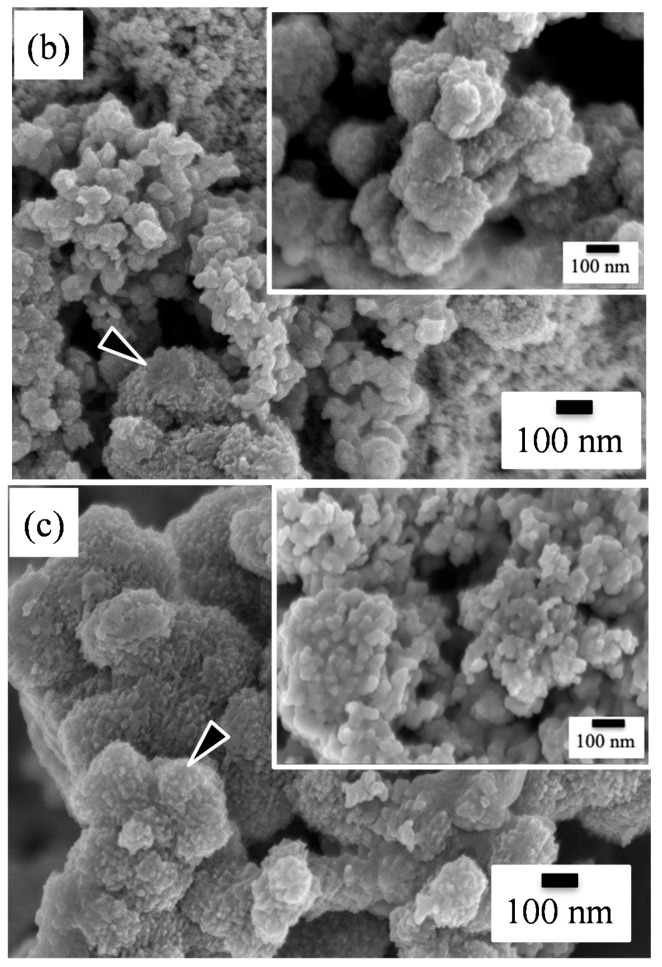
SEM micrographs of the screen-printed SnO_2_ powder coatings (**a**) without, and with (**b**) 45 and (**c**) 55 wt % PANI deposition after heat treatment. The insets in the images (**b**,**c**) show the corresponding higher magnification SEM images.

**Figure 4:**
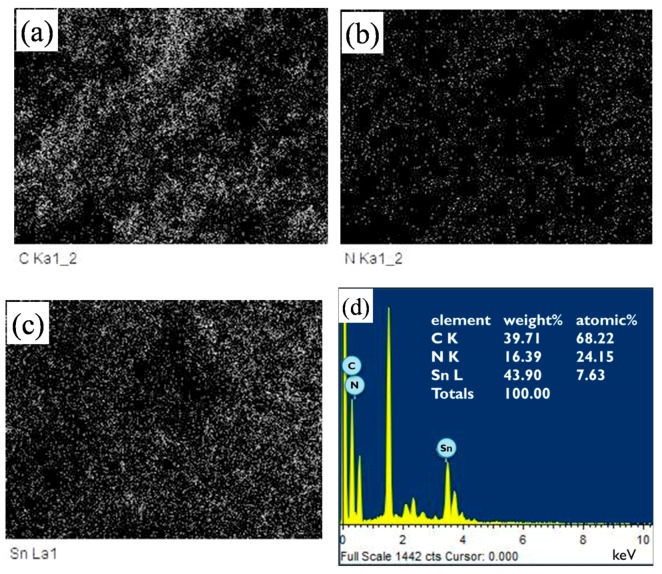
EDS mappings of (**a**) C, (**b**) N, and (**c**) Sn obtained from a screen-printed SnO_2_ powder coating with 45 wt % PANI deposition. (**d**) EDS spectrum and elemental analysis of the 45 wt % deposited SnO_2_ coating.

Figure 3 presents the SEM micrographs of the as-screen printed SnO_2_ powders without and with PANI deposition. As shown in Figure 3a, the morphology of the SnO_2_ powder coatings without PANI deposition exhibited a structure of nanoparticle distribution after heat treatment. From the SEM image, the particle size in the SnO_2_ coating was estimated to be 8 ± 2 nm with a relatively narrow distribution, being very close to the crystallite sizes calculated from the XRD patterns. This suggests that each SnO_2_ particle was made up of a single crystallite. Compared to the particle size of the as-synthesized SnO_2_ powder, the size in the powder coating increased slightly after heat treatment. Generally, a gas-sensing material with a smaller particle size has a higher sensor performance [29,30,31]. As shown in Figure 3b,c, the drop-coated PANI particles on top of the SnO_2_ coatings were observed to be cluster-like agglomerates with a spherical morphology, as indicated by the arrows. With PANI deposition, the agglomeration of the nanoparticles in the powder coatings tended to become more severe, although there was no significant difference in the particle sizes of the SnO_2_ particle coatings with and without PANI deposition. This suggests that the nanoparticle agglomerates formed while the PANI nanoparticles were drying. Furthermore, the morphology of the SnO_2_ coatings exhibited shrunken nanoparticle agglomeration, resulting in a porous structure. Such a porous structure provides a higher specific surface area and is considered to be advantageous for gas sensing properties [27,30]. Figure 4 shows the EDS (energy dispersive x-ray spectroscopy) mappings and spectrum of a SnO_2_ powder coating with 45 wt % PANI deposition in the SEM image of Figure 3b. Note that in Figure 4a–c, the EDS dot-mapping analyses of C, N, and Sn confirmed the existence of PANI and tin dioxide in the sensor coating. The SnO_2_ powder coating was uniformly deposited by PANI. The EDS spectrum in Figure 4d also implies the successful synthesis of the PANI/SnO_2_ composite coating. The data in the insert in Figure 4d list the elemental composition of the PANI/SnO_2_ powder coating from the EDS analysis. The elements of C and N were quantitatively analyzed, showing that the atomic ratio of C/N was about 3/1. This ratio suggests that the deposited polymer on the SnO_2_ coating was composed of PANI (C_12_H_12_N_4_). 

**Figure 5:**
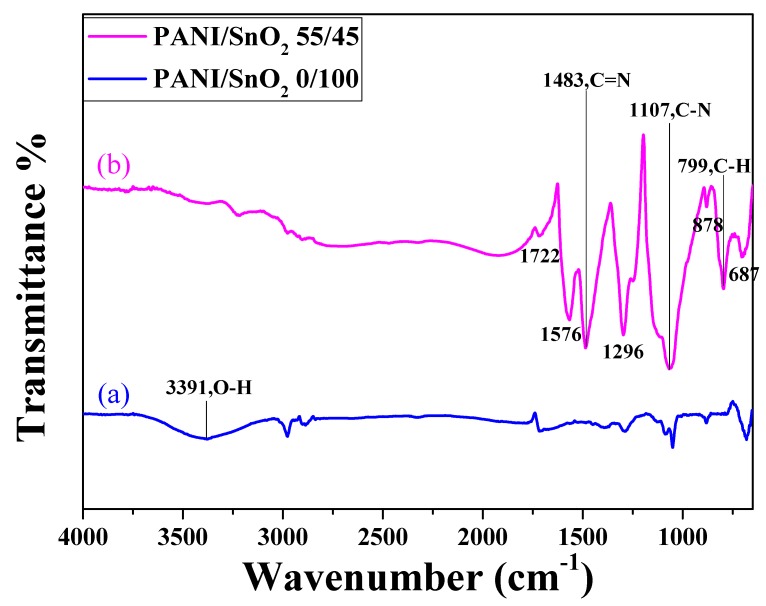
FTIR spectra of the SnO_2_ powder coatings (**a**) without and (**b**) with 55 wt % PANI deposition.

Figure 5 shows the FTIR spectra of the SnO_2_ powder coatings without and with PANI deposition. The FTIR spectrum of the SnO_2_ coating with PANI deposition (curve (b)) was almost identical to that of PANI reported in the literature [32,33,34], unlike the spectrum of the undeposited one (curve (a)). Note that in curve (b), the two bands at approximately 1576 and 1483 cm^−1^ were due to the C–C stretching vibration of the quinoid and benzenoid rings, respectively [32]. The band at approximately 1296 cm^−1^ was associated with the C–N mode, while the peak observed at approximately 1107 cm^−1^ was the characteristic band of the stretching vibration of quinoid, and the presence of a band at approximately 799 cm^−1^ was attributed to the out-of-plane deformation of C–H in a benzene ring [33,34]. This demonstrates that the PANI was successfully deposited onto the surface of the SnO_2_ coating. 

**Figure 6:**
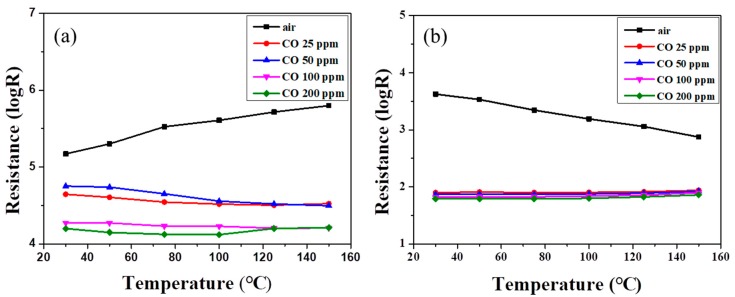

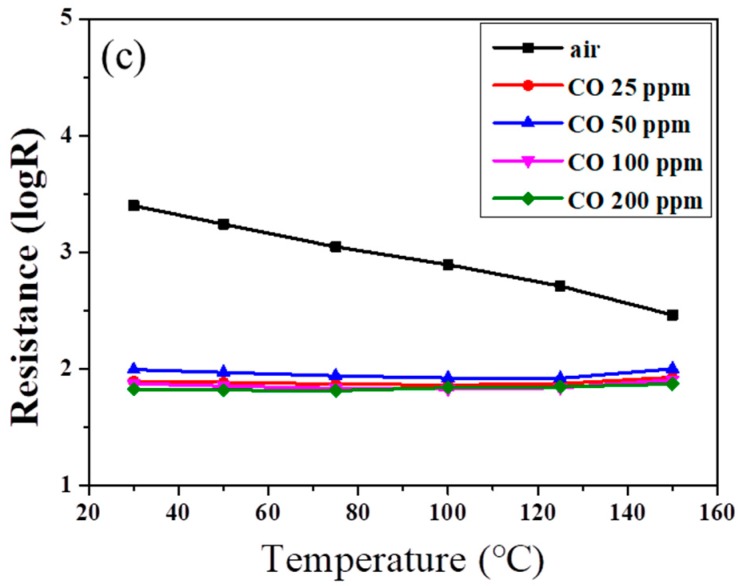
Electrical resistance of screen-printed SnO_2_ powder coatings (**a**) without, and with (**b**) 45, and (**c**) 55 wt % PANI depositions under different CO concentrations as a function of sensing temperature.

**Figure 7:**
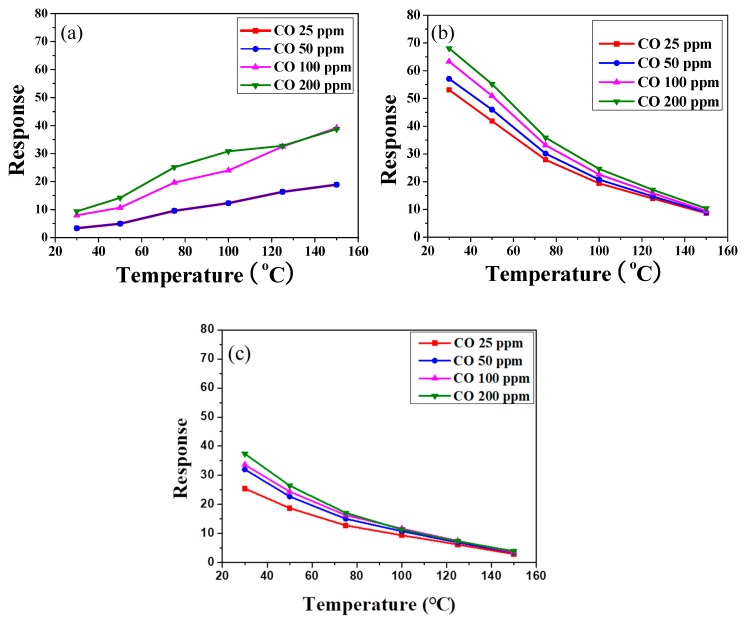
Sensor response of the screen-printed SnO_2_ powder coatings (**a**) without, and with (**b**) 45 and (**c**) 55 wt % PANI depositions under different CO concentrations as a function of sensing temperature.

Figure 6 shows the resistances of SnO_2_ powder coatings with different amounts of PANI deposition under various CO concentrations as a function of sensing temperature. Note that all the SnO_2_ sensors exhibited lower resistance at higher CO partial pressure, exhibiting *n*-type sensing behavior [1,35]. In Figure 6a, the resistance of the powder coating without PANI deposition increased slightly with increases in air sensing temperature until the temperature reached 150 °C. Similar trends were found in our earlier work for sensing temperatures below 200 °C [11]. However, in an atmosphere containing CO gas, the resistance of the SnO_2_ coating showed no obvious change with the increase in sensing temperature. All resistances exhibited a similar trend as the sensing temperature was elevated under different CO concentrations. Figure 7 shows the sensor response, *R*_air_/*R*_gas_, of the SnO_2_ coatings with various amounts of PANI deposition under different CO concentrations as a function of sensing temperature. The different trends in resistance between air and CO gas may cause an enhancement of the sensor response of an undeposited SnO_2_ sensor at higher temperature, as shown in Figure 7a. Meanwhile, the sensor response was lower than 25 at low sensing temperature (<75 °C). Furthermore, returning to Figure 6b,c, the resistances of SnO_2_ coatings with PANI deposition decreased linearly with increases in the air sensing temperature. Compared with the SnO_2_ coating without PANI, the resistances of PANI-deposited SnO_2_ coatings significantly decreased in CO gas. However, no obvious change was found under different CO concentrations as the sensing temperature increased. That is, the deposition of PANI (Figure 7b,c) decreased the resistance of SnO_2_ in CO gas at a lower sensing temperature, enhancing the difference in resistance of the SnO_2_ coating under air and CO gas. The sensor response of SnO_2_ with 45 wt % PANI deposition was thus significantly increased under low CO concentration at sensing temperatures below 75 °C, as shown in Figure 7b. The sensor response of the PANI/SnO_2_ composite sensors ranged from ca. 40 to 70 at operating temperatures of 30–50 °C. Such values are comparable to those reported in the literature [36].

**Figure 8:**
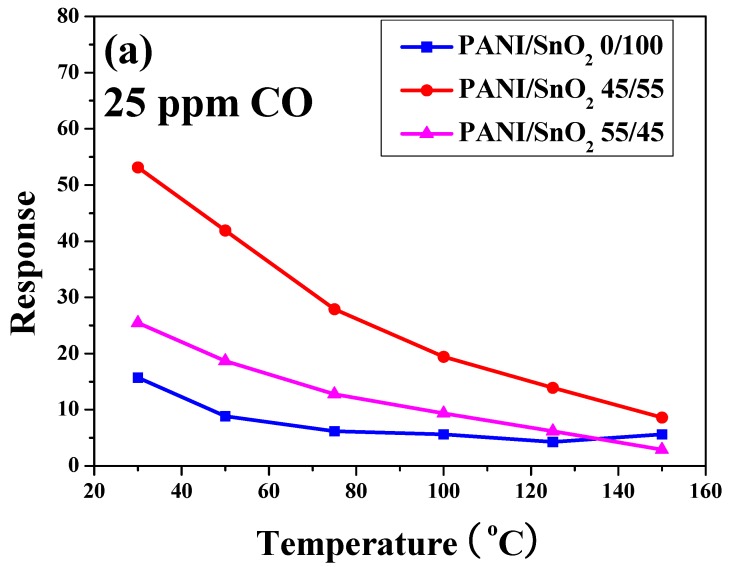

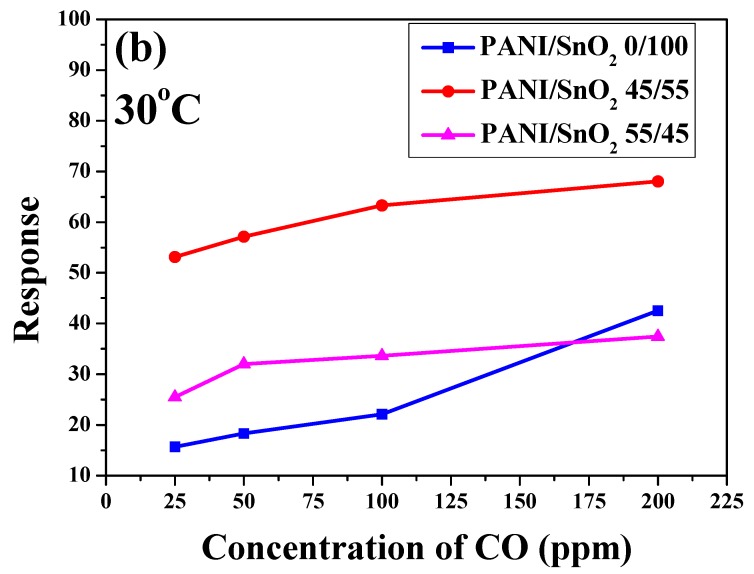
Sensor response of the screen-printed SnO_2_ powder coatings without and with PANI deposition as functions of (**a**) operating temperature and (**b**) CO concentration.

To further understand the influence of temperature and CO concentration on sensing behavior, the sensor responses of the SnO_2_ powder coatings with and without PANI deposition as functions of operating temperature and CO concentration were plotted for comparison (Figure 8). As shown in Figure 8a, the sensor response of all of the SnO_2_ powder coatings decreased with increases in operating temperature under a CO concentration of 25 ppm. Similar trends can also be found in the same PANI/SnO_2_ composite system reported in the literature [31]. The SnO_2_ powder coating without PANI deposition exhibited the lowest sensor response. Note that the powder coating with 45 wt % PANI deposition possessed the highest sensor response in this system, showing the highest value of 53 even under a CO concentration of 25 ppm at 30 °C. This indicates that the PANI deposition improved the sensor response of the SnO_2_ powder coating to CO gas at low working temperature. Furthermore, in Figure 8b, the sensor response of all of the SnO_2_ powder coatings increased with increases in CO concentration at an operating temperature of 30 °C. As mentioned previously, the SnO_2_ powder coating with 45 wt % PANI deposition exhibited the highest sensor response under all of the different CO concentrations in this system. For SnO_2_ based materials, the sensing mechanism has been explained using the space-charge layer model and band model [17,31,37,38]. 

**Figure 9:**
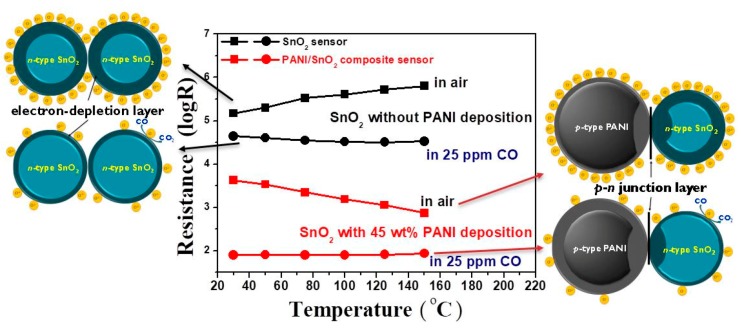
Resistance of the SnO_2_ powder coatings without and with 45 wt % PANI deposition as a function of operating temperature under air and 25 ppm CO. The corresponding schematic diagrams based on the space-charge layer model of the sensors are also shown in this figure.

Figure 9 shows the resistance of the SnO_2_ powder coatings without and with 45 wt % PANI deposition under air and 25 ppm CO, respectively. As PANI is a *p*-type semiconductor, two competitive mechanisms of electronic properties occur in the PANI/SnO_2_ composite coating when PANI is deposited onto an *n*-type SnO_2_ semiconductor [39]. Their corresponding schematic diagrams based on the space-charge layer model are also shown in this figure. When the SnO_2_ coating without PANI deposition is exposed to clean air, the oxygen molecules adsorbed on the SnO_2_ surface extract electrons to form oxygen ion species and cause the formation of an electron-depleted layer in the SnO_2_ surface. Such a so-called space-charge layer reduces the carrier concentration of the materials, causing a very high resistance of the SnO_2_ powder coating. When the SnO_2_ powder coating is exposed to CO gas, reaction of those adsorbed oxygen ion species with CO gas releases electrons to increase the carrier concentration, causing a resistance reduction of the SnO_2_ powder coating, as shown in the diagram on the left side of Figure 9. With the deposition of conductive PANI, however, the resistances of the PANI/SnO_2_ composite coating under both air and CO gas decreased significantly. Note that the variation of resistances of the composite coating in air and CO increased in the low temperature region. This increase can be attributed to the wider depletion layers caused by the formation of a *p*–*n* junction at the interface between the PANI and SnO_2_ materials. When the PANI/SnO_2_ sensor is exposed to air, the resistance decreases with increases in operating temperature, exhibiting the sensing behavior of an *n*-type semiconductor. This implies that the sensing mechanism of PANI/SnO_2_ is dominated by SnO_2_. When the PANI/SnO_2_ sensor is exposed to CO gas, the *p*–*n* junction can lower the activation energy and enthalpy of physisorption for vapors with good electron-donating characteristics [31,40]. The composite sensor composed of conductive PANI and SnO_2_ with thin depletion layers (as shown in the diagram on the right side of Figure 9) exhibited a temperature-independent low resistance. The sensor response was thus increased by depositing PANI on SnO_2_ from a response value of 15 to 53 in 25 ppm CO at 30 °C.

**Figure 10:**
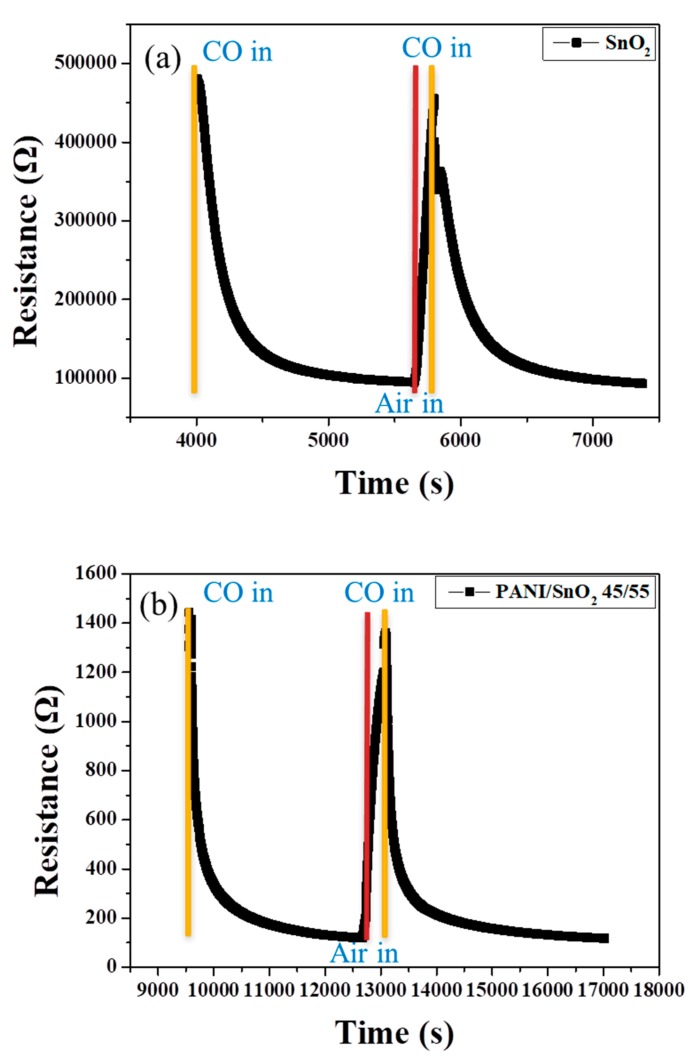
Dynamic sensor response of SnO_2_ powder coatings (**a**) without and (**b**) with 45 wt % PANI deposition as a function of time toward a change in CO concentration in the range of air to 50 ppm at 100 °C.

Figure 10 shows the corresponding dynamic sensor response to the change in CO concentration of the SnO_2_ coating without and with PANI deposition as a function of time at 100 °C. Note that the undeposited SnO_2_ coating showed an obvious response to a change in CO concentration in the range of air to 50 ppm, as shown in Figure 10a. When the atmosphere returned to air, the resistance of the SnO_2_ coating recovered rapidly, showing a relatively good sensing behavior at an operating temperature of 100 °C. This may have resulted from the relatively low heat-treatment temperature and extremely small particle size of the SnO_2_ powder coating. The structure of the powder coating, influenced by the particle size, was thought to be crucial to the sensing performance. With 45 wt % PANI deposition onto the surface of the SnO_2_ powder coating, as shown in Figure 10b, the response toward a change in CO concentration increased significantly at low sensing temperatures, being more than three times higher than that of the SnO_2_ coating with no PANI deposition (Figure 7b). Similarly, the PANI-deposited SnO_2_ coating showed relatively good sensing reversibility and stability. The most likely reason is the good properties of the deposited PANI for redox reaction during sensing, which caused the higher difference in resistance of the composite sensor in oxidizing (in air) and reducing atmospheres (in CO), as shown in Figure 6b. This effect increased the sensor response, *R*_air_/*R*_gas_, at low sensing temperature in this system. Furthermore, response time (*t*_90_) is defined as the time when the ratio (*R*_air_ − *R*)/(*R*_air_ − *R*_gas_) becomes 0.9 after the 50 ppm CO gas suddenly reacts with the sensor. The response times for the SnO_2_ powder coatings without and with 45 wt % PANI deposition were estimated to be ca. 510 s and ca. 720 s, respectively. It was found that the response time of the SnO_2_ coating was not shortened by PANI deposition. The likely reason is that the porous structure that allows the CO gas to enter into the SnO_2_ coating may have been blocked by the PANI deposition during sensing, although the PANI-deposited SnO_2_ coating showed a better sensor response performance. The details of the sensing mechanism still need to be further clarified in the future.

## 4. Conclusions

Sol-gel SnO_2_ nanosized powder was screen-printed onto Al_2_O_3_ substrates and different amounts of PANI were deposited by drop-coating for the investigation of the CO sensor properties. The resulting powder was identified as a tetragonal SnO_2_ phase and observed to have an extremely small particle size of ca. 5–9 nm. The screen-printed SnO_2_ coating revealed a uniformly loose structure and still had a very small crystallite size of 6–10 nm after heat treatment. With PANI deposition, the morphology of the SnO_2_ powder coating exhibited nanoparticle agglomeration with a porous structure. The deposition of conductive PANI tended to cause a greater difference in the resistance of the composite sensor between air and CO gas at low sensing temperature. Toward all the concentrations (25–200 ppm) of CO gas in this system, the sensor response of PANI-deposited SnO_2_ sensors ranged from ca. 40 to 70 at operating temperatures of 30–50 °C, showing a good sensor response toward the change in CO concentration at low temperature. The dynamic sensor response of the PANI/SnO_2_ composite sensors also exhibited relatively good reversibility, indicating excellent sensing stability.
